# Hydroponically Grown *Sanguisorba minor* Scop.: Effects of Cut and Storage on Fresh-Cut Produce

**DOI:** 10.3390/antiox8120631

**Published:** 2019-12-09

**Authors:** Costanza Ceccanti, Marco Landi, Gabriele Rocchetti, Maria Begoña Miras Moreno, Luigi Lucini, Luca Incrocci, Alberto Pardossi, Lucia Guidi

**Affiliations:** 1 Department of Agriculture, Food and Environment, University of Pisa, 56124 Pisa, Italy; c.ceccanti3@studenti.unipi.it (C.C.); luca.incrocci@unipi.it (L.I.); alberto.pardossi@unipi.it (A.P.); lucia.guidi@unipi.it (L.G.); 2 Interdepartmental Research Center Nutrafood “Nutraceuticals and Food for Health”, University of Pisa, 56124 Pisa, Italy; 3 Department for Sustainable Food Process, Università Cattolica del Sacro Cuore, 29121 Piacenza, Italy; Gabriele.Rocchetti@unicatt.it (G.R.); mariabegona.mirasmoreno@unicatt.it (M.B.M.M.); luigi.lucini@unicatt.it (L.L.)

**Keywords:** metabolomic profiling, functional food, salad burnet, storage, wild edible species

## Abstract

Wild edible plants have been used in cooking since ancient times. Recently, their value has improved as a result of the scientific evidence for their nutraceutical properties. *Sanguisorba minor* Scop. (salad burnet) plants were hydroponically grown and two consecutive cuts took place at 15 (C1) and 30 (C2) days after sowing. An untargeted metabolomics approach was utilized to fingerprint phenolics and other health-related compounds in this species; this approach revealed the different effects of the two cuts on the plant. *S. minor* showed a different and complex secondary metabolite profile, which was influenced by the cut. In fact, flavonoids increased in leaves obtained from C2, especially flavones. However, other secondary metabolites were downregulated in leaves from C2 compared to those detected in leaves from C1, as evidenced by the combination of the variable important in projections (VIP score > 1.3) and the fold-change (FC > 2). The storage of *S. minor* leaves for 15 days as fresh-cut products did not induce significant changes in the phenolic content and antioxidant capacity, which indicates that the nutraceutical value was maintained. The only difference evidenced during storage was that leaves obtained from C2 showed a lower constitutive content of nutraceutical compounds than leaves obtained from C1; except for chlorophylls and carotenoids. In conclusion, the cut was the main influence on the modulation of secondary metabolites in leaves, and the effects were independent of storage.

## 1. Introduction

Since ancient times, many wild edible plants have been used in cooking. Recently, their value has improved thanks to their proven nutraceutical properties [1,2,3]. In fact, some researchers have recognized wild edible plants as functional foods and as a new source of bioactive compounds that are beneficial to human health for their anti-inflammatory, antimicrobic, anticarcinogenic, cytotoxic and antiproliferative properties [4,5,6,7,8,9]. For example, a number of wild plants have been used in the diet including the stems and leaves of *Sanguisorba minor*, the fruit of *Rosa canina,* bellota acorns of *Quercus ilex* [5], leaves of *Umbelicus rupestris* (Salisb.) Dandy [10] and wild edible flowers [11].

Salad burnet (*S. minor* Scop.) is a wild edible species traditionally known for its edibility, and use in folk medicine, nowadays, it is also recognized for its potential as a nutraceutical species [12]. This wild edible species is a perennial herb of the *Rosaceae* family distributed in the Sinai Peninsula, Egypt, as well as in temperate areas in Europe. It has been used in traditional medicine because of its hypoglycemic activity, which is caused by triterpenes (23-hydroxytormentic acid ester glucoside and 23-hydroxytormentic acid) [13,14]. This species is also able to inhibit the inflammatory processes in which radical oxygen species are over-produced, such as the inflammatory processes associated with Alzheimer’s disease, because of the inhibition of the glycogen synthase kinase 3β, which plays an important role in Alzheimer’s disease [15,16]. 

The medicinal and functional properties of this species are linked to its high antioxidant activity and the high content of bioactive compounds. In fact, Ranfa et al. [17] showed that this species had the highest total polyphenolic content (258 mg 100 g^−1^) compared to other commonly utilized wild edible plants. Vanzani et al. [18] also confirmed a very high amount of polyphenols in salad burnet. Furthermore, high levels of α-tocopherol (85 mg kg^−1^ dry matter) and β-carotene (30 mg kg^−1^ dry matter) have been found in this species [19].

Many other bioactive compounds, such as aliphatic hydrocarbons, sesquiterpenes, farnesyl acetate, nonadecane, β-carotene, vitamin E, vitamin C and phenols sensu lato has been isolated from this species, and research is ongoing [12,13,14]. In addition, many compounds with antioxidant activity such as 1-O-β-galloyl-glucose, 2,3-hexahydroxydiphenoyl-(α/β)-glucose, gallic acid, 1-galloyl-2,3-hexahydroxy-diphenoyl-α-glucose and its β-isomer, quercetin-3-O-β-(6”-galloylgalactoside, kaempferol, quercetin, ellagic acid, 4,8-dimethoxy-7-hydroxy-2-oxo-2H-1-benzopyran-5,6-dicarboxylic acid, and 2-(4-carboxy-3-methoxystyryl)-2-methoxysuccinic acid have been isolated from salad burnet [13]. 

According to traditional recipes, the leaves of the young plants of salad burnet can be used in mixed salads [12]. In our society, the use of this species as fresh-cut produce is interesting since minimally processed vegetables are one of the top ten fastest growing consumed foods in the world [20]. The introduction of a new leafy species with demonstrated nutraceutical properties as fresh-cut produce will help to increase the availability of different foods in the Mediterranean diet. Furthermore, several studies on the effect of storage of some species that are widely used as fresh-cut produce have shown that the polyphenolic content and antioxidant activity are not affected [21,22]. 

Therefore, a deep metabolomic characterization of this promising leafy species is necessary in order to unveil new, potentially interesting compounds that improve human health. In addition, an evaluation of the main nutraceutical metabolite classes upon storage could help consumers and producers to understand the potential of this species to be merchandised as “new” fresh-cut produce.

In a preliminary experiment, it was observed that salad burnet was a wild edible species with high adaptability, which allows it to be cultivated in hydroponics systems [3]. In this report, we investigated firstly, the metabolomic characterization of salad burnet leaves from two consecutive cuts, which was then analyzed in order to find new compounds for this species that have never been reported in the literature; and secondly, we evaluated the effect of storage, thus the pattern of the main nutraceutical compounds (e.g., phenols, chlorophylls, carotenoids and ascorbic acid) was monitored during the storage of the fresh-cut salad burnet leaves derived from both of the cuts. 

## 2. Materials and Methods

### 2.1. Plant Materials and Growth Conditions

Seedlings of salad burnet were cultivated in a floating system at the University of Pisa in a greenhouse during the period from 15 June to 17 July 2018. The growing conditions were: 31 °C average temperature, 50.9% humidity and 128.1 W m^−2^ light intensity. The plants were grown in a nutritive solution composed of: NO_3_^−^ 10 mM, NH_4_^+^ 0.5 mM, PO_4_^3−^ 1 mM, K^+^ 6 mM, Ca^2+^ 4 mM, Mg^2+^ 2 mM, Na^+^ 0.5 mM, SO_4_^2−^ 3.5 mM, Cl^−^ 0.5 mM, HCO_3_^−^ 0.5 mM, Fe^2+^ 40 µM, BO_3_^−^ 25 µM, Cu^2+^ 1 µM, Zn^2+^ 5 µM, Mn^2+^ 10 µM, Mo^3+^ 1 µM. Electrical conductivity was 1.98 dS m^−1^; pH was adjusted to 5.7–6.0 with diluted sulphuric acid. The nutrient solution was continuously aerated. The plants were grown for 15 days after sowing and when plants had approximately 20 leaves, leaves over 5 cm were cut off at the base (C1). The same plants re-grew and after a further 15 days (30 days after sowing), leaves over 5 cm were cut off at the base (C2). After both cuts, leaves were sampled for metabolomics analyses.

A portion of the leaves from both of the cuts were also processed as fresh-cut produce and stored at 4 °C in dark conditions in polyethylene tetraphtalate (PET) boxes (150 cm^3^, Comital Cofresco, Italy). Each box contained approximately 15 g. After being stored, the fresh-cut products were sampled (after 1, 2, 3, 6, 9, 13 and 15 days) to analyze their phenol, flavonoid and ascorbic acid content and the antioxidant activity.

### 2.2. Extraction and Untargeted Metabolomics-Based Profiling of Fresh Plant Material Obtained from Two Consecutive Cuts

Samples of the leaves derived from the two cuts were utilized for the extraction of secondary metabolites through a homogenizer-assisted extraction (Ultra-turrax; Ika T25, Staufen, Germany), according to Borgognone et al. [23]. A total of 1 g of leaves were homogenized in 10 mL of 80% methanol solution (*v/v*) acidified with 0.1% formic acid. The extracts were centrifuged at 6000 *g* for 15 min at 4 °C. The resulting solutions were filtered using 0.22 µm cellulose syringe filters in dark vials and stored at −18 °C until analysis.

Each sample was analyzed in triplicate by ultra-high-pressure liquid chromatography (UHPLC) coupled with quadrupole-time-of-flight (QTOF) mass spectrometry (Agilent Technologies, Santa Clara, CA, USA). The experimental conditions for the screening of secondary metabolites in different plant matrices were optimized in previous work [24]. Briefly, the mass spectrometer was set to operate in SCAN mode, acquiring positive ions from a JetStream electrospray source (ESI +) in the range of 100–1200 m/z. The chromatographic separation was achieved in the reverse phase mode using an Agilent Zorbax Eclipse Plus C18 column (100 × 2.1 mm, 1.8 μm) and a mixture of water (phase A) and acetonitrile (phase B) as the mobile phase (both liquid chromatography-mass spectrometry grade). Besides, formic acid 0.1% (*v/v*) (Sigma-Aldrich, Milan, Italy) was added to both phases. The gradient went from 6% acetonitrile to 94% acetonitrile within 35 min, the flow rate was 0.22 mL min^−1^ and the injection volume was 6 μL.

Raw data were then processed using the software Agilent Profinder B.07 and the “find-by-formula” algorithm, thus combining monoisotopic accurate mass and isotopic profile [25]. A custom database containing both phenolics (as reported in Phenol-Explorer 3.6; phenol-explorer.eu/) and sesquiterpene lactones was built and used as a reference for annotation, adopting a 5-ppm tolerance for mass accuracy. Some other compounds, which were reported as characteristic in the plant species targeted, were also mined in our raw data using the above-mentioned approach (Supplementary Materials). The annotation of secondary metabolites was carried out according to a LEVEL 2 of accuracy, as set out by the Metabolomics Standard Initiative [26].

Following annotation, the phenolic compounds were ascribed to different classes (according to Phenol-Explorer) and quantified using methanolic standard solutions prepared from an individual reference compound per each class, as previously reported [27].

These analyses were effectuated in fresh material after the first and second cut of the plants to characterize the profile of secondary metabolites in the salad burnet.

### 2.3. Samples Preparation for Phytochemical Analysis of Stored Material

Leaves were taken during storage (after 1, 2, 3, 6, 9, 13 and 15 days), homogenized and frozen in liquid nitrogen, and stored at −80 °C for biochemical analyses. 

### 2.4. Pigment Analysis 

Spectrophotometric analysis of pigments was performed by an Ultrospec 2100 Pro spectrophotometer (GE Healthcare Ltd., Little Chalfont, England) following the method described by Porra et al. [28], with minor modifications. Fresh samples (0.3 g) were extracted in 20 mL of acetone 80% and agitated in the dark at 4 °C for 3 days. The chlorophyll and carotenoid content were determined by the increase in absorbance at 663 nm for chlorophyll *a*, 648 nm for chlorophyll *b* and 470 nm for carotenoids against a blank solution of acetone 80%. Total chlorophylls and carotenoids were expressed as mg g^−1^ fresh weight (FW).

### 2.5. Phenol and Flavonoid Extraction

Samples (about 1 g FW) were homogenized with 4 mL of methanol solution 80% (*v/v*) by a sonicator (Digital ultrasonic Cleaner, DU-45, Argo-Lab, Modena, Italy) for 30 min, keeping the temperature in the range of 0–4 °C. Samples were centrifuged through a centrifuge (MPW-260R, MWP Med. Instruments, Warsaw, Poland) at 10,000 *g* for 15 min at 4 °C and supernatants were collected and centrifuged again in a 2 mL Eppendorf tube for 3 min at 7000 *g.* Extracts were stored at −80 °C before analysis.

### 2.6. Total Phenolic Determination

Total phenolic content was measured according to the procedure described by Dewanto et al. [29] with minor modifications. Briefly, 10 μL extract samples were mixed with 125 μL Folin-Ciocalteu reagent and 115 μL distilled water and allowed to react for 6 min. Then, 1.25 mL of Na_2_CO_3_ 7% (*w/v*) were added and samples were incubated for 90 min in the dark at room temperature. The increase in absorbance at 760 nm was measured against a blank solution (without sample). The results were expressed as mg gallic acid equivalents per g FW (mg GAE g^−1^ FW).

### 2.7. Flavonoid Determination

Total flavonoid content was determined according to Du et al. [30] with minor modifications. In a 2 mL Eppendorf tube, 100 μL sample extracts were added to 400 μL distilled water and 30 μL NaNO_2_ 5% (*w/v*). After 6 min at room temperature, 30 μL AlCl_3_·6H_2_O 0.3 M and 30 μL distilled water were added to the mixture. After 6 min at room temperature, 400 μL NaOH 4% (*w/v*) and 40 μL distilled water were added to the extract. The increase in absorbance at 515 nm was measured against a blank solution (which contained all the reagents without extract). Total flavonoid content was expressed as mg catechin equivalents per g FW (mg CAE g^−1^ FW).

### 2.8. In Vitro Antioxidant Activity Analysis

In vitro antioxidant activity was determined on the same extract that was utilized for phenol and flavonoid analyses by using the 2,2-diphenyl-1-picrylhydrazyl (DPPH) free radical scavenging assay, as described by Brand-Williams et al. [31] with minor modifications. Sample extracts of 10 μL were added to 990 μL of DPPH solution 3.12 × 10^−^^5^ M and incubated in the dark for 30 min at room temperature. The decrease in absorbance at 515 nm was measured against a blank solution (without extract). The results were expressed as mg Trolox equivalent antioxidant capacity per g FW (mg TEAC g^−1^ FW).

### 2.9. Ascorbic Acid Extraction

Fresh leaves (about 0.3 g FW) were homogenized with 6% (*w/v*) trichloric acetic acid (TCA). After sample centrifugation (10,000 *g* for 10 min at 4 °C), the supernatant was collected in 2 mL Eppendorf tubes. 

### 2.10. Total Ascorbic Acid Analysis

Total ascorbic acid (ASA) was determined spectrophotometrically as described by Kampfenkel et al. [32]. Briefly, 50 μL of extract was incubated at 42 °C for 15 min with 50 μL dithiothreitol (DTT) 10 mM and with 100 μL Na-P buffer 0.2 M (pH 7.4). After 15 min, 50 μL N-ethylmaleimide (NEM) 0.5% (*w/v*) was added and samples were stirred for 1 min. TCA (250 μL; 6% (*w/v*)), 200 μL H_3_PO_4_ 42% (*w/v*), 200 μL 2,2’-dipyridil 4% (*w/v*) (diluted in ethanol 70% (*v/v*)) and 100 μL FeCl_3_ 3% (*w/v*) were added to the samples and incubated at 42 °C for 40 min. The increase in absorbance at 525 nm was measured against a blank solution (without sample). Ascorbic acid was expressed as mg ascorbic acid on g FW (mg ASA g^−1^ FW).

### 2.11. Statistical Analysis

Data are the mean ± standard deviation (SD) of 3 replicates in each assay. Biochemical data were analyzed by two-way analysis of variance (ANOVA) using storage and cut as sources of variation; the means were separated by Fisher’s least significant difference (LSD) *post-hoc* test (*p* = 0.05). All statistical analyses were conducted using GraphPad (GraphPad, La Jolla, CA, USA) or the statistical software PASW Statistics 25.0 (SPSS Inc., Chicago, IL, USA).

Metabolomics data were elaborated using the software Agilent Mass Profiler Professional B.12.06, as previously reported [27]. Compounds were filtered by abundance (only those compounds with an area > 5000 counts were considered), normalized at the 75th percentile and baselined to their corresponding median. Post-acquisition processing also included filtering by frequency, and retaining those compounds identified within 100% of replications in at least one treatment. Unsupervised hierarchical cluster analysis (HCA) was then carried out using the Euclidean similarity measure and Wards as the linkage rule. Thereafter, the metabolomic dataset was exported into SIMCA 13 (Umetrics, Malmo, Sweden), Pareto scaled and elaborated for supervised orthogonal partial least squares discriminant analysis (OPLS-DA). The presence of outliers was excluded according to Hotelling’s T2, while cross-validation of the model was done using analysis of variance of the cross-validated residuals (CV-ANOVA) (*p* < 0.01) and permutation testing (*N* = 100). For each OPLS-DA built, the model parameters (goodness-of-fit (R^2^Y) and goodness-of-prediction (Q^2^Y)) were also inspected. Moreover, the variables importance in projection (VIP) compounds selection approach was applied (VIP > 1.3) and combined with a fold-change analysis (FC > 2) in order to evaluate those secondary metabolites significantly affected by cut.

## 3. Results and Discussion

### 3.1. UHPLC-QTOF Mass Spectrometry Untargeted Profiling and Effect of the Two Cuts

The secondary metabolites profile in leaves from both cuts of the salad burnet was investigated by using untargeted metabolomics (UHPLC-QTOF mass spectrometry) to depict the changes in the main secondary metabolites induced by the cuts. Overall, the untargeted approach allowed us to putatively identify 467 compounds in the leaves of *S. minor*. A list of the identified secondary metabolites is reported in the Supplementary Materials, together with the composite mass spectra (Table S1). The analyses confirmed the richness in polyphenols in this species as observed in previous research [17,18]. Annotated polyphenols were then ascribed to distinct phenolic classes, and then the changes between the two cuts were investigated (Table S2).

The content of all flavonoid sub-classes increased in the leaves from C2 and flavones was the sub-class with the highest increase (FC value = 1.4). Among flavones, apigenin, baicalein and 7,3’,4’-trihydroxyflavone were the most representative. Apigenin is abundant in common fruits such as grapefruit and orange, and vegetables such as onion and parsley [33]. The biological activity of this flavone in numerous mammalian systems is related to its antioxidant effects and its role in free radicals scavenging. However, it also shows anti-mutagenic, anti-inflammatory, antiviral and purgative effects [34]. The most important effect of this flavone is related to cancer prevention, because it induces apoptosis in human cancer cells and has an anti-proliferative effect on human cancer cells [33]. Apigenin has also demonstrated other positive features for human health, such as the reduction of plasma levels of low-density lipoproteins and the inhibition of platelet aggregation, thus, its presence in the diet could be very important [33,34,35]. Other studies have shown that baicalein can mitigate cell proliferation and diminish the production of collagen; also, it possesses multiple effective properties for the treatment of different diseases including cancer and injuries [36,37,38,39]. Lastly, 7,4’-dihydroxyflavone, also named 5-deoxyluteolin, has shown impressive antimycobacterial activity and its anti-inflammatory activity is under evaluation [40]. The comparison of C1 versus C2 leaves showed an increase in anthocyanins and flavones, although with moderate FC values.

No significant variations were observed for the other phenolic classes, as reported in the Supplementary Materials (Table S2).

**Figure 1:**
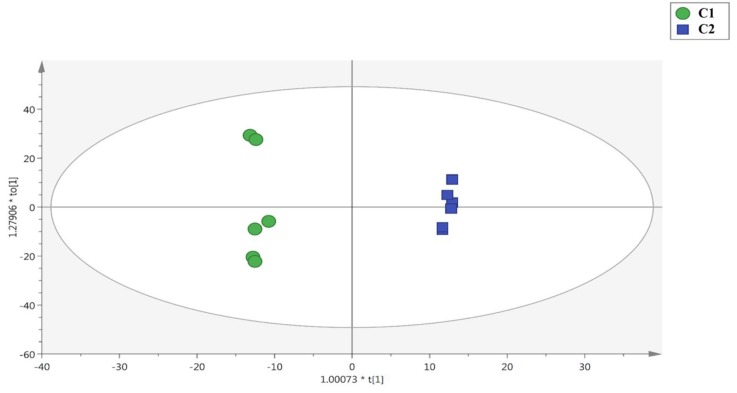
Orthogonal projection to latent structures (OPLS) discriminant analysis (DA) on secondary metabolites characterizing *Sanguisorba minor* at first (C1) and second cut (C2).

Subsequently, both unsupervised and supervised multivariate statistical approaches were used to further investigate the differences in secondary metabolites in relation to the cut. The outputs of the unsupervised HCA heat map are provided in the Supplementary Materials (Table S3). The HCA heat maps highlighted the clear modification of the leaf secondary metabolite profiles in relation to the cut. Therefore, in order to explore these differences, supervised OPLS-DA modeling was used to underline those metabolites that were responsible of these variations. Interestingly, it was evident that the model clearly discriminated the two cuts, as shown in Figure 1.

The second latent vector of the OPLS-DA score plot was found to discriminate the two cuts accurately. The model showed high prediction ability, with a Q^2^Y parameter of 0.69. Besides, the OPLS-DA model was cross-validated and tested for outliers. Therefore, the variable selection method (VIP) was used to identify the compounds that were most responsible for the different leaf secondary metabolite profiles. These compounds with a VIP score higher than 1.3, are reported in Table S4 together with their LogFC value for the pairwise comparison of leaves from the second cut versus the first cut. Secondary metabolites possessing a FC value > 2 were selected for this purpose. A high number of compounds effectively showed the differences between the two cuts (i.e., 76 secondary metabolites, including mainly flavonoids, phenolic acids and sesquiterpene lactones) (Table S4). The compounds with the highest LogFC values, when considering the upregulated category, were the phenolic acid avenanthramide 2p (LogFC = 14.2) followed by a sesquiterpene lactone (i.e., artemissifolin; LogFC = 13.9). Phenolic acids have been shown to exert powerful biological activity [41,42,43]. Among avenanthramides, which are characteristic in oat [41,42,43,44,45,46,47], avenanthramide 2p modulates problematic events in β-catenin mediated transcriptional activation of Wnt target gene (fundamental for the survival of cells), the c-MYC proto-oncogene, and in this way reduces the proliferation of cervical cancer cells in vitro [48]. Concerning artemissifolin, this sesquiterpene is mainly found in the genus *Centaurea* [49,50], but its biological activity still needs to be clarified.

Three compounds showed the highest downregulation values when compared to the others, being 3,4-diferuloylquinic acid (a chlorogenic acid; LogFC = −17.6), epicebellin J (a guaianolide; LogFC = −13.8) and desacetyl-beta-cyclopyrethrosin (a sesquiterpene lactone; LogFC = −13.2). The 3,4-diferuloylquinic acid was isolated in the genus *Coffea* [51] and it is reported that high amounts of chlorogenic acids can inhibit the proliferation of cancer cells due to their biological activity in the human colon [52,53]. Epicebellin J was found in *Centaurea glastifolia* [54] and its nutraceutical activity is unknown; conversely, guaianolides can have antitumor, anti-inflammatory, and antibacterial activities [55]. The last downregulated compound was the desacetyl-β-cyclopyrethrosin, which has antibacterial activity, especially against Gram-negative bacteria [56].

In conclusion, the metabolomic screening of salad burnet leaves allowed us to identify new organic compounds that have not previously been reported in this species. In addition, leaves from the second cut exhibited an overall lower level of the main representative compounds, except for flavonoids and, in particular, flavones.

### 3.2. Phytochemical Analyses in Leaves Stored as Fresh-Cut Product

In order to generate knowledge about changes in the main nutraceutical compounds (chlorophylls, carotenoids, total phenols, flavonoids and ascorbic acid) and the total antioxidant activity of salad burnet leaves obtained from both the cuts, time-course measurements were carried out during the storage period (15 days) of the fresh-cut produce.

#### 3.2.1. Pigments

**Figure 2:**
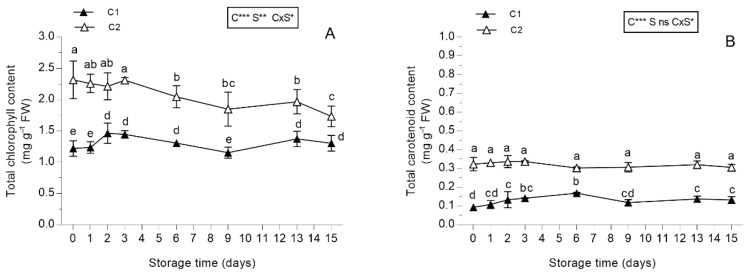
Total chlorophyll and total carotenoid contents of *Sanguisorba minor* (**A**,**B**), respectively) stored at 4 °C for 15 days as fresh-cut produce. Closed and open symbols represent the first (C1) and second cut (C2), respectively. Each value is the mean ± standard deviation of 3 replicates. Means keyed with the same letter are not significantly different for *p* = 0.05 following two-way analysis of variance (ANOVA) with storage (S) and cut (C) as variability factors. ns: not significant; *: *p* < 0.05; **: *p* < 0.01; ***: *p* < 0.001 for each factor and their interaction.

Cut had a strong effect and a significant increase in chlorophyll content was detected in leaves from the second cut (Figure 2A). At harvest time, the difference in chlorophyll content between C1 and C2 was 53%, but this difference decreased after cold storage of the fresh-cut produce (29.5% at the end of the storage). This was attributable to the steep decline in chlorophyll detected in leaves obtained from C2. In leaves derived from C1, the chlorophyll content during storage was more stable than that detected in leaves from C2 (Figure 2A). Moreover, the trend in the chlorophyll content of this species after storage was much higher than that reported in lettuce species, which are widely used as fresh-cut produce [49]. In fact, the chlorophyll content in lettuce was 0.4, 0.4, and 0.3 mg g^−1^ FW after 0, 7, and 14 days of storage, respectively; these values are much lower than those recorded in *S. minor* [57].

Similar to chlorophyll, carotenoid content was also higher in leaves derived from C1 than those from C2, and it was higher than that reported in fresh-cut lettuce species in cold storage [58]. Furthermore, the pattern of the carotenoid content remained unchanged during storage in salad burnet leaves obtained from C1, whereas a slight increment was observed in C2 leaves during storage (Figure 2B). However, carotenoid content decreased in lettuce after it was stored for 10 days [58].

#### 3.2.2. Total Phenolic Content (TPC)

**Figure 3:**
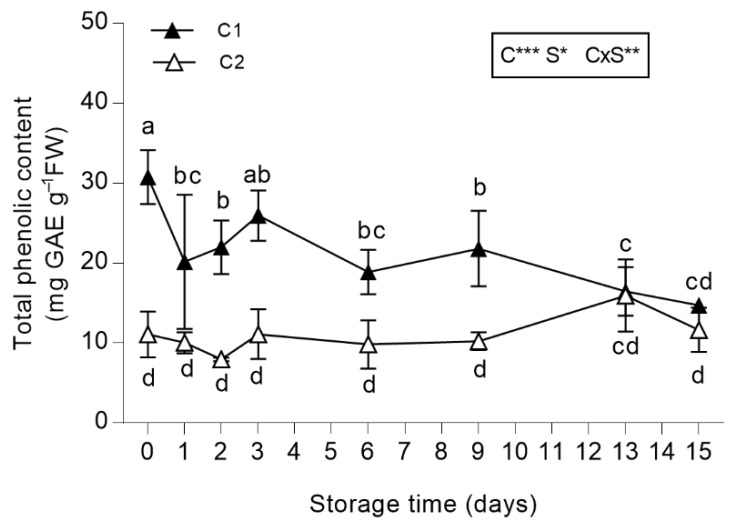
Total phenolic content (TPC) of *Sanguisorba minor* stored at 4 °C for 15 days as fresh-cut produce. Closed and open symbols represent first (C1) and second cut (C2), respectively. Each value is the mean ± standard deviation of 3 replicates. Means keyed with the same letter are not significantly different for *p* = 0.05 following two-way analysis of variance (ANOVA) with storage (S) and cut (C) as variability factors. *: *p* < 0.05; **: *p* < 0.01; ***: *p* < 0.001 for each factor and their interaction. TPC values were expressed as gallic acid equivalents (GAE) mg g^−1^ FW.

At harvest, the phenolic content in salad burnet leaves largely exceeded the TPC measured in lettuce species [59,60]. In particular, TPC was found to be higher in the leaves from the first cut (32.4 mg g^−1^ FW) than in the leaves from the second cut (being 22.7 mg g^−1^ FW; *p* < 0.01). These results are also in agreement with the cumulative amounts of phenols that were found using the UHPLC-QTOF semi-quantitative approach (Table S5). During the storage, TPC decreased almost regularly in leaves from C1, even though the greatest reduction was observed on the first day of storage (Figure 3). Moreover, TPC in leaves obtained from C2 remained mostly constant during storage (Figure 3).

The pattern for the leaves obtained from C2 was close to that found in lettuce, escarole and rocket salad during their storage as fresh-cut produce, even though the values of TPC were higher in *S. minor* than in those leafy vegetables (approximately 0.9, 0.10 and 4.5 μg g^−1^ FW in lettuce, escarole and rocket salad, respectively) [61]. Therefore, the TPC of *S. minor* was higher than that found in leafy species that are widely used as fresh-cut produce [61,62,63].

#### 3.3.3. Total Flavonoid Content

**Figure 4:**
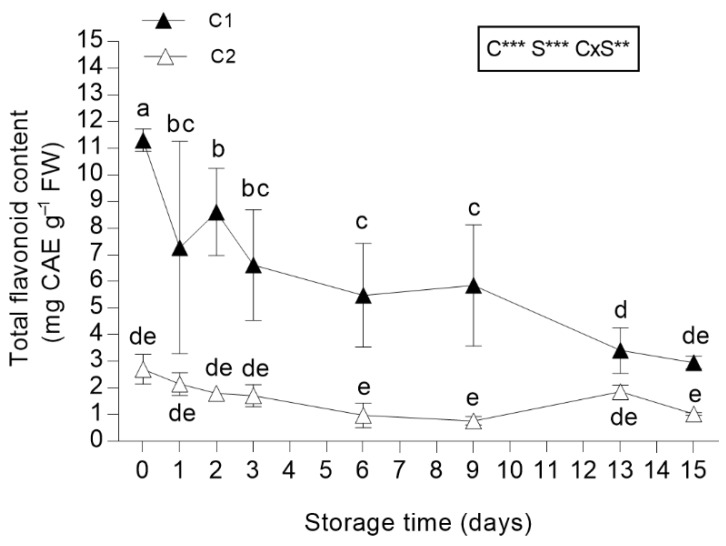
Total flavonoid content in *Sanguisorba minor* stored at 4 °C for 15 days as fresh-cut produce. Closed and open symbols represent first (C1) and second cut (C2), respectively. Each value is the mean ± standard deviation of 3 replicates. Means keyed with the same letter are not significantly different for *p* = 0.05 following two-way analysis of variance (ANOVA) with storage (S) and cut (C) as variability factors. ns: not significant; **: *p* < 0.01; ***: *p* < 0.001 for each factor and their interaction. Total flavonoid content values were expressed as catechin equivalents (CAE) mg g^−1^ FW.

The flavonoid content in salad burnet leaves was higher in leaves belonging to C1 as compared to those recorded in leaves from C2 (+76.1%; Figure 4). Storage had a negative impact on C1 leaves. In fact, the total flavonoid content strongly decreased after just one day of storage, as total phenol content, but unlike these last compounds, the flavonoid content declined until the end of the storage period (Figure 4). Notably, high variability was observed among samples from 1 to 9 days of storage. Differently, the total flavonoid content in leaves from C1 remained stable during storage (Figure 4). The total flavonoid content denotes the powerful nutraceutical value of this species, since many leafy species that are widely used as fresh-cut produce show lower total flavonoid content [64,65,66]. In fact, authors have recorded 0.25 mg CAE g^−1^ FW for lettuce Canasta, 0.15 mg CAE g^−1^ FW for chicory Catalogna, and 0.29 mg CAE g^−1^ FW for chicory Spadona, all lower values than those found for salad burnet leaves [65].

#### 3.3.4. Total Ascorbic Acid (ASA) Content

**Figure 5:**
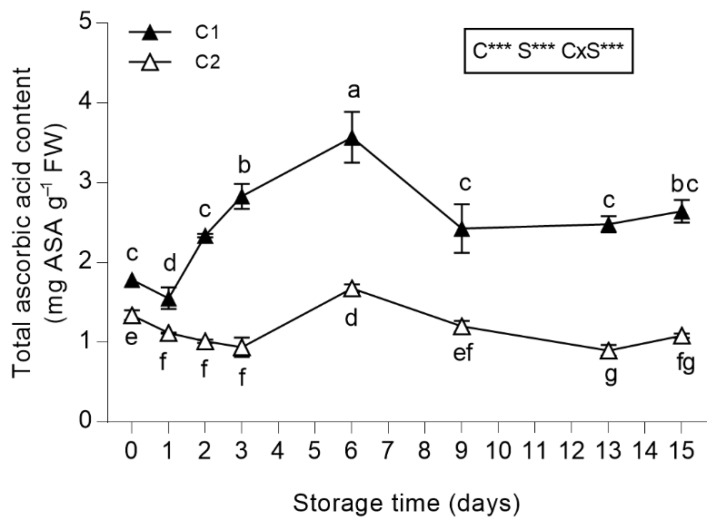
Total ascorbic acid (ASA) content of *Sanguisorba minor* stored at 4 °C for 15 days as fresh-cut produce. Closed and open symbols represent first (C1) and second cut (C2), respectively. Each value is the mean ± standard deviation of 3 replicates. Means keyed with the same letter are not significantly different for *p* = 0.05 following two-way analysis of variance (ANOVA) with storage (S) and cut (C) as variability factors. ***: *p* < 0.001 for each factor and their interaction.

The leaves from C1 contained higher levels of ASA as compared to those from C2 (Figure 5). In leaves obtained from C1, ASA content increased significantly until the sixth day of storage when it reached 3.57 mg g^−1^ FW (Figure 5). Then, a decrease in ASA content was recorded, and at the end of the storage period, it was similar to the values detected at harvest. Conversely, ASA content in leaves from C2 had a more constant trend (Figure 5). However, the ASA content of salad burnet leaves is higher than that found in lettuce species that are widely used as fresh-cut produce [67,68]. In fact, Barry-Ryan and O’Beirne [67] reported 0.25 mg ASA g^−1^ FW in iceberg lettuce and Bonasia et al. [68] found 0.15 mg ASA g^−1^ FW in rocket; lower values than those found for salad burnet leaves.

#### 3.3.5. In Vitro Total Antioxidant Activity

**Figure 6:**
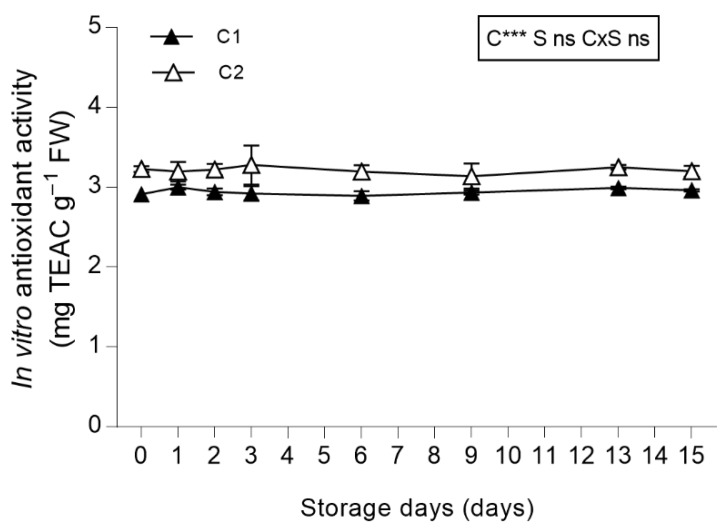
In vitro antioxidant activity of *Sanguisorba minor* stored at 4 °C for 15 days as fresh-cut produce. Closed and open symbols represent first (C1) and second cut (C2), respectively. Each value is the mean (± SD) of 3 replicates. Means keyed with the same letter are not significantly different for *p* = 0.05 following two-way analysis of variance (ANOVA) with storage (S) and cut (C) as variability factors. ns: not significant; ***: *p* < 0.001 for each factor and their interaction. The lack of letters denotes the lack of significance in the interaction of the variability factors. In vitro antioxidant activity values were expressed as Trolox equivalent antioxidant capacity (TEAC) mg g^−1^ FW.

In vitro antioxidant activity was higher in leaves from C2 than those from C1, whereas no effects were recorded in relation to their storage as fresh-cut produce (Figure 6).

The in vitro antioxidant capacity in leaves from both of the cuts and during storage was higher (3.23 mg TEAC g^−1^ FW in C1 leaves at harvest) than the in vitro antioxidant activity reported by other authors in leafy species widely used as fresh-cut produce [64,69,70]. Khanam et al. [64] reported values of in vitro antioxidant capacity determined by DPPH assay that averaged 0.69 mg TEAC g^−1^ FW in iceberg lettuce, 0.99 in Canasta lettuce, 2.44 in Continental lettuce and 1.01 in escarole. Viacava et al. [69] reported 0.75 mg TEAC g^−1^ FW in Butterhead lettuce, and Mampholo et al. [70] reported 0.4 mg TEAC g^−1^ FW in green lettuce varieties. For this reason, salad burnet leaves show promise as powerful antioxidant fresh-cut produce.

## 4. Conclusions

*Sanguisorba minor* showed a complex secondary metabolite profile that was influenced by the cut. Flavonoid content increased in leaves obtained from C2, especially the sub-class of flavones, which is characterized by anti-mutagenic, antioxidant, anti-inflammatory, anti-viral and purgative effects and is also highly important in cancer prevention [33,34,35,36,37,38,39]. Moreover, secondary metabolites were downregulated in leaves from C2 as compared to those detected in leaves from C1, as evidenced by the combination of the VIP score (VIP > 1.3) and the fold-change (FC > 2).

During their storage as fresh-cut produce, salad burnet leaves did not show any remarkable changes in their phenolic profile and antioxidant capacity, a positive result for the maintenance of the nutraceutical value, especially considering the high levels of these compounds as compared to other leafy species that are widely used as fresh-cut produce [53,54,59,60,61,62,63,64,65]. Also, the content of ascorbic acid, a pivotal antioxidant compound, strongly increased in the first days (until the third day) of storage and remained higher than values recorded in leafy species widely used as fresh-cut produce [67,68]. The only differences evidenced during storage were the lower constitutive content (T0) of nutraceutical compounds in leaves obtained from C2 than in leaves obtained from C1, except for chlorophylls and carotenoids. In conclusion, the cut was the main influence on the modulation of secondary metabolites in leaves, independently of the storage.

This work shows that pre- and post-harvest factors can influence the nutraceutical value of product. The results show that the *S. minor* wild edible species is rich in bioactive compounds that are maintained during their storage as fresh-cut produce. The results suggest that this species could be a valid alternative to other leafy species commonly utilized for salad preparation, and also a promising species in a world in which food and flavor industries require new food ingredients for food supplements. Future research is necessary to investigate the possibility of increasing the nutraceutical content of this species by agronomical factors, for example, by managing the nutrient solution in hydroponic systems, and how to increase the stability of the nutraceutical values of *S. minor* between consecutive cuts.

## Supplementary Materials

The following are available online at https://www.mdpi.com/2076-3921/8/12/631/s1, Table S1: Secondary metabolites and their composite mass spectra, Table S2: Phenolic classes, Table S3: HCA heat map, Table S4: Comparison C2 *versus* C1 (VIP score and Log FC values), Table S5: Amount of phenols sub-classes.

## References

[1] Romojaro A., Botella M.A., Obón C., Pretel M.T. (2013). Nutritional and antioxidant properties of wild edible plants and their use as potential ingredients in the modern diet. Int. J. Food Sci. Nutr..

[2] Morales P., Ferreira I.C., Carvalho A.M., Sánchez-Mata C., Cámara M., Fernández-Ruiz V., Pardo-de-Santayana M., Tardío J. (2014). Mediterranean non-cultivated vegetables as dietary sources of compounds with antioxidant and biological activity. Food Sci. Technol..

[3] Ceccanti C., Landi M., Benvenuti S., Pardossi A., Guidi L. (2018). Mediterranean wild edible plants: Weeds or “new functional crops”?. Molecules.

[4] Vallverdú-Queralt A., Regueiro J., Martínez-Huélamo M., Rinaldi Alvarenga J.F., Leal L.N., Lamuela-Raventos R.M. (2014). A comprehensive study on the phenolic profile of widely used culinary herbs and spices: Rosemary, thyme, oregano, cinnamon, cumin and bay. Food Chem..

[5] Sanchez-Bel P., Romojaro A., Egea I., Pretel M.T. (2015). Wild edible plants as potential antioxidant or nutritional supplements for beverages minimally processed. LWT-Food Sci. Technol..

[6] Petropoulos S.A., Karkanis A., Martins N., Ferreira I.C.F.R. (2018). Edible halophytes of the Mediterranean basin: Potential candidates for novel food products. Trends Food Sci. Technol..

[7] Umate S.K., Marathe V.R. (2018). Nutraceutical evaluation of *Acalypha indica* L.—A potential wild edible plant. Int. J. Green Pharm..

[8] Tanase C., Mocan A., Coșarcă S., Gavan A., Nicolescu A., Gheldiu A.M., Vodnar D.C., Muntean D.L., Crișan O. (2019). Biological and chemical insights of beech (*Fagus sylvatica* L.) bark: A source of bioactive compounds with functional properties. Antioxidants.

[9] Coșarcă S.L., Moacă E.A., Tanase C., Muntean D.L., Pavel I.Z., Dehelean C.A. (2019). Spruce and beech bark aqueous extracts: Source of polyphenols, tannins and antioxidants correlated to in vitro antitumor potential on two different cell lines. Wood Sci. Technol..

[10] Iyda J.H., Fernandes Â., Calhelha R.C., Alves M.J., Ferreira F.D., Barros L., Amaral J.S., Ferreira I.C. (2019). Nutritional composition and bioactivity of Umbilicus rupestris (Salisb.) Dandy: An underexploited edible wild plant. Food Chem..

[11] Pires T.C., Barros L., Santos-Buelga C., Ferreira I.C. (2019). Edible flowers: Emerging components in the diet. Trends Food Sci. Technol..

[12] Guarrera P.M., Savo V. (2016). Wild food plants used in traditional vegetable mixtures in Italy. J. Ethnopharmacol..

[13] Ayoub N.A. (2003). Unique phenolic carboxylic acids from *Sanguisorba minor*. Phytochemistry.

[14] Reher G., Slijepcevié M., Kraus L. (1991). Hypoglycemic activity of triterpenes and tannins from *Sarcopoterium spinosum* and two *Sanguisorba* species. Planta Med..

[15] Ferreira A., Proença C., Serralheiro M.L.M., Araújo M.E.M. (2006). The in vitro screening for acetylcholinesterase inhibition and antioxidant activity of medicinal plants from Portugal. J. Ethnopharmacol..

[16] Kaufmann D., Herrmann F., Wink M. (2009). Extracts from traditional Chinese medical plants inhibit glycogen synthase kinase 3 β activity, a potential Alzheimer target. Z. Phytother..

[17] Ranfa A., Maurizi A., Romano B., Bodesmo M. (2014). The importance of traditional uses and nutraceutical aspects of some edible wild plants in human nutrition: The case of Umbria (central Italy). Plant Biosyst..

[18] Vanzani P., Rossetto M., De Marco V., Sacchetti L.E., Paoletti M.G., Rigo A. (2011). Wild Mediterranean plants as traditional food: A valuable source of antioxidants. J. Food Sci..

[19] Elgersma A., Søegaard K., Jensen S.K. (2013). Fatty acids, α-tocopherol, β-carotene, and lutein contents in forage legumes, forbs, and a grass-clover mixture. J. Agric. Food Chem..

[20] Manzocco L., Foschia M., Tomasi N., Maifreni M., Dalla Costa L., Marino M., Cortella G., Cesco S. (2011). Influence of hydroponic and soil cultivation on quality and shelf life of ready-to-eat lamb’s lettuce (*Valerianella locusta* L. Laterr). J. Sci. Food Agric..

[21] Landi M., Ruffoni B., Salvi D., Savona M., Guidi L. (2015). Cold storage does not affect ascorbic acid and polyphenolic content of edible flowers of a new hybrid of sage. Agrochimica.

[22] Landi M., Ruffoni B., Combournac L., Guidi L. (2018). Nutraceutical value of edible flowers upon cold storage. Ital. J. Food Sci..

[23] Borgognone D., Rouphael Y., Cardarelli M., Lucini L., Colla G. (2016). Changes in biomass, mineral composition, and quality of cardoon in response to NO_3_^−^:Cl^−^ ratio and nitrate deprivation from the nutrient solution. Front. Plant Sci..

[24] Paul K., Sorrentino M., Lucini L., Rouphael Y., Cardarelli M., Bonini P., Moreno M.B.M., Reynaud H., Canaguier R., Trtílek M. (2019). A combined phenotypic and metabolomic approach for elucidating the biostimulant action of a plant-derived protein hydrolysate on tomato grown under limited water availability. Front. Plant Sci..

[25] Rocchetti G., Lucini L., Chiodelli G., Giuberti G., Montesano D., Masoero F., Trevisan M. (2017). Impact of boiling on free and bound phenolic profile and antioxidant activity of commercial gluten-free pasta. Food Res. Int..

[26] http://cosmos-fp7.eu/msi.

[27] Rocchetti G., Bhumireddy S.R., Giuberti G., Mandal R., Lucini L., Wishart D.S. (2019). Edible nuts deliver polyphenols and their transformation products to the large intestine: An in vitro fermentation model combining targeted/untargeted metabolomics. Food Res. Int..

[28] Porra R.J., Thompson W.A., Kriedemann P.E. (1989). Determination of accurate extinction coefficients and simultaneous equations for assaying chlorophylls a and *b* extracted with four different solvents: Verification of the concentration of chlorophyll standards by atomic absorption spectroscopy. Biochim. Biophys. Acta Biogenrg..

[29] Dewanto V., Adom K.K., Liu R.H. (2002). Thermal processing enhances the nutritional value of tomatoes by increasing total antioxidant activity. J. Agric. Food Chem..

[30] Du G., Li M., Ma F., Liang D. (2009). Antioxidant capacity and the relationship with polyphenol and vitamin C in Actinidia fruits. Food Chem..

[31] Brand-Williams W., Cuvelier M.E., Berset C. (1995). Use of a free radical method to evaluate antioxidant activity. LWT-Food Sci. Technol..

[32] Kampfenkel K., Van Montagu M., Inzé D. (1995). Extraction and determination of ascorbate and dehydroascorbate from plant tissue. Anal. Biochem..

[33] Shukla S., Gupta S. (2010). Apigenin: A promising molecule for cancer prevention. Pharm. Res..

[34] Yang C.S., Landau J.M., Huang M.T., Newmark H.L. (2001). Inhibition of carcinogenesis by dietary polyphenolic compounds. Annu. Rev. Nutr..

[35] O’Prey J., Brown J., Fleming J., Harrison P.R. (2003). Effects of dietary flavonoids on major signal transduction pathways in human epithelial cells. Biochem. Pharmacol..

[36] Liu J., Wan J., He C.W. (2010). Rationale for the use of natural anti-inflammatory agents in cancer chemotherapy. N. Am. J. Med. Sci..

[37] Wang L., Ling Y., Chen Y., Li C.L., Feng F., You Q.D., Lu N., Guo Q.L. (2010). Flavonoid baicalein suppresses adhesion, migration and invasion of MDA-MB-231 human breast cancer cells. Cancer Lett..

[38] Yang L., Li X., Zhang S., Song J., Zhu T. (2019). Baicalein inhibits proliferation and collagen synthesis of mice fibroblast cell line NIH/3T3 by regulation of miR-9/insulin-like growth factor-1 axis. Artif. Cells Nanomed. Biotechnol..

[39] Chokchaisiri R., Suaisom C., Sriphota S., Chindaduang A., Chuprajob T., Suksamrarn A. (2009). Bioactive flavonoids of the flowers of *Butea monosperma*. Chem. Pharm. Bull..

[40] Marques J., Silva A.M.S., Marques M.P.M., Braga S.S. (2019). Ruthenium(II) trithiacyclononane complexes of 7,3′,4′-trihydroxyflavone, chrysin and tectochrysin: Synthesis, characterisation, and cytotoxic evaluation. Inorganica Chim. Acta.

[41] Multari S., Pihlava J.M., Priscilla Ollennu-Chuasam P., Hietaniemi V., Yang B., Suomela J.P. (2018). Identification and quantification of avenanthramides and free and bound phenolic acids in eight cultivars of husked oat (*Avena sativa* L.) from Finland. J. Agric. Food Chem..

[42] Escobedo-Flores Y., Chavez-Flores D., Salmeron I., Molina-Guerrero C., Perez-Vega S. (2018). Optimization of supercritical fluid extraction of polyphenols from oats (*Avena sativa* L.) and their antioxidant activities. J. Cereal Sci..

[43] de Bruijn W.J.C., van Dinteren S., Gruppen H., Vincken J.P. (2019). Mass spectrometric characterization of avenanthramides and enhancing their production by germination of oat (*Avena sativa*). Food Chem..

[44] Collins F.W. (1989). Oat Phenolics: Avenanthramides, novel substituted N-cinnamoylanthranilate alkaloids from oat groats and hulls. J. Agric. Food Chem..

[45] Chen C.Y.O., Milbury P.E., Collins F.W., Blumberg J.B. (2007). Avenanthramides are bioavailable and have antioxidant activity in humans after acute consumption of an enriched mixture from oats. J. Nutr..

[46] Perrelli A., Goitre L., Salzano A.M., Moglia A., Scaloni A., Retta S.F. (2018). Biological activities, health benefits, and therapeutic properties of avenanthramides: From skin protection to prevention and treatment of cerebrovascular diseases. Oxidative Med. Cell. Longev..

[47] Bratt K., Sunnerheim K., Bryngelsson S., Fagerlund A., Engman L., Andersson R.E., Dimberg L.H. (2003). Avenanthramides in oats (*Avena sativa* L.) and structure-antioxidant activity relationships. J. Agric. Food Chem..

[48] Wang D., Wise M.L., Li F., Dey M. (2012). Phytochemicals attenuating aberrant activation of β-catenin in cancer cells. PLoS ONE.

[49] Gousiadou C., Skaltsa H. (2003). Secondary metabolites from *Centaurea orphanidea*. Biochem. Syst. Ecol..

[50] Grafakou M.E., Djeddi S., Tarek H., Skaltsa H. (2018). Secondary metabolites from the aerial parts of *Centaurea papposa* (Coss.) Greuter. Biochem. Syst. Ecol..

[51] Perrone D., Farah A., Donangelo C.M., de Paulis T., Martin P.R. (2008). Comprehensive analysis of major and minor chlorogenic acids and lactones in economically relevant Brazilian coffee cultivars. Food Chem..

[52] Olthof M.R., Hollman P.C.H., Katan M.B. (2001). Chlorogenic acid and caffeic acid are absorbed in humans. J. Nutr..

[53] Kahle K., Huemmer W., Kempf M., Scheppach W., Erk T., Richling E. (2007). Polyphenols are intensively metabolized in the human gastrointestinal tract after apple juice consumption. J. Agric. Food Chem..

[54] Ӧksüz S., Topcu G. (1994). Guaianolides from *Centaurea glastifolia*. Phytochemistry.

[55] Ma G.H., Chen K.X., Zhang L.Q., Li Y.M. (2019). Advance in biological activities of natural guaiane-type sesquiterpenes. Med. Chem. Res..

[56] Konstantinopoulou M., Karioti A., Skaltsas S., Skaltsa H. (2003). Sesquiterpene lactones from *Anthemis altissima* and their anti-*Helicobacter pylori* activity. J. Nat. Prod..

[57] Perucka I., Olszówka K., Chilczuk B. (2013). Changes in the chlorophyll content in stored lettuce *Lactuca sativa* L. after pre-harvest foliar application of CaCl_2_. Acta Agrobot..

[58] Hill L.E., Oliveira D.A., Hills K., Giacobassi C., Johnson J., Summerlin H., Taylor T.M., Gomes C.L. (2017). A Comparative study of natural antimicrobial delivery systems for microbial safety and quality of fresh-cut lettuce. J. Food Sci..

[59] Materska M., Olszówka K., Chilczuk B., Stochmal A., Pecio Ł., Pacholczyk Sienicka B., Piacente S., Pizza C., Masullo M. (2019). Polyphenolic profiles in lettuce (*Lactuca sativa* L.) after CaCl_2_ treatment and cold storage. Eur. Food Res. Technol..

[60] Riga P., Benedicto L., Gil-Izquierdo Á., Collado-González J., Ferreres F., Medina S. (2019). Diffuse light affects the contents of vitamin C, phenolic compounds and free amino acids in lettuce plants. Food Chem..

[61] Degl’Innocenti E., Pardossi A., Tognoni F., Guidi L. (2007). Physiological basis of sensitivity to enzymatic browning in ‘lettuce’, ‘escarole’ and ‘rocket salad’ when stored as fresh-cut products. Food Chem..

[62] Altunkaya A., Gökmen V. (2008). Effect of various inhibitors on enzymatic browning, antioxidant activity and total phenol content of fresh lettuce (*Lactuca sativa*). Food Chem..

[63] Beltrán D.M., Selma M.V., Marín A., Gil M.I. (2005). Ozonated water extends the shelf life of fresh-cut lettuce. J. Agric. Food. Chem..

[64] Khanam U.K.S., Oba S., Yanase E., Murakami Y. (2012). Phenolic acids, flavonoids and total antioxidant capacity of selected leafy vegetables. J. Funct. Foods.

[65] Heimler D., Vignolini P., Arfaioli P., Isolania L., Romani A. (2012). Conventional, organic and biodynamic farming: Differences in polyphenol content and antioxidant activity of Batavia lettuce. J. Sci. Food Agric..

[66] Pérez-López U., Sgherri C., Miranda-Apodaca J., Micaelli F., Lacuesta M., Mena-Petite A., Quartacci M.F., Muñoz-Rueda A. (2018). Concentration of phenolic compounds is increased in lettuce grown under high light intensity and elevated CO_2_. Plant Physiol. Biochem..

[67] Berry-Ryan C., O’Beirne D. (1999). Ascorbic acid retention in shredded iceberg lettuce as affected by minimal processing. J. Food Sci..

[68] Bonasia A., Lazzizera C., Elia A., Conversa G. (2017). Nutritional, biophysical and physiological characteristics of wild rocket genotypes as affected by soilless cultivation system, salinity level of nutrient solution and growing period. Front. Plant Sci..

[69] Viacava G.E., Goyeneche R., Goñi M.G., Roura S.I., Agüero M.V. (2018). Natural elicitors as preharvest treatments to improve postharvest quality of Butterhead lettuce. Sci. Hortic..

[70] Mampholo B.M., Maboko M.M., Soundy P., Sivakumar D. (2016). Phytochemicals and overall quality of leafy lettuce (*Lactuca sativa* L.) varieties grown in closed hydroponic system. J. Food Qual..

